# Mild to moderate frailty among older adults does not affect long‐term quality of life or functional outcomes after colon cancer surgery

**DOI:** 10.1111/codi.70438

**Published:** 2026-04-07

**Authors:** Maria Normann, Eva Haglind, Peter Matthiessen, Jacob Rosenberg, Niklas Ekerstad, Eva Angenete, Mattias Prytz

**Affiliations:** ^1^ Department of Surgery, Institute of Clinical Sciences, Sahlgrenska Academy University of Gothenburg Gothenburg Sweden; ^2^ Department of Surgery NU‐Hospital Group Trollhättan Sweden; ^3^ Department of Surgery, SSORG – Scandinavian Surgical Outcomes Research Group, Institute of Clinical Sciences, Sahlgrenska Academy University of Gothenburg Gothenburg Sweden; ^4^ Department of Surgery Sahlgrenska University Hospital Gothenburg Sweden; ^5^ Faculty of Medicine and Health Sciences, Department of Surgery Örebro University Örebro Sweden; ^6^ Department of Surgery Örebro University Hospital Örebro Sweden; ^7^ Department of Surgery, Herlev Hospital University of Copenhagen Copenhagen Denmark; ^8^ Department of Health, Medicine and Caring Sciences Linköping University Linköping Sweden; ^9^ Department of Research and Development NU‐Hospital Group Trollhättan Sweden

**Keywords:** colorectal cancer, frailty, outcome, QoL

## Abstract

**Background:**

Frailty is known to adversely affect post‐operative mortality and morbidity following colorectal cancer resection, but its impact on long‐term quality of life and functional outcomes after colon cancer surgery is less clear. This study aims to evaluate the impact of frailty at the time of diagnosis on quality of life, impact on daily activities, and contentment with treatment one year after colon cancer resection.

**Method:**

Data were obtained from the prospective, multicentre Quality of Life in Colon Cancer (QoLiCOL) study. Patients aged ≥70 years who underwent colon cancer surgery in Region Västra Götaland, Sweden, were collected from the QoLiCOL database (*n* = 347). Clinical data were retrieved from national quality registries. Frailty was retrospectively assessed by reviewing medical records using the Clinical Frailty Scale‐9 (CFS‐9), with scores ≥4 classified as frail. Outcomes included self‐reported quality of life, treatment‐related impact on activities of daily living, and treatment satisfaction one year post‐operatively. Directed Acyclic Graphs (DAGs) of variables known to affect the outcome variables were made before analyses, and potential confounders were adjusted for in the final analyses. Comparisons between frail and non‐frail groups were performed using ordinal logistic regression and logistic regression, with results reported as odds ratios (OR).

**Results:**

The prevalence of frailty in the cohort was 29%, with a median CFS‐9 value of 4 (range 4–6) in the frail group, indicating very mild to moderate frailty. No significant differences were observed between frail and non‐frail older adults in self‐assessed quality of life, treatment‐related impact on daily activities or treatment satisfaction one year after surgery. Notably, both groups reported improved quality of life at one year compared with baseline. Only a small proportion of participants (*n* = 8; 2%) reported not being content with their treatment.

**Conclusion:**

Among older adults who were alive one year after colon cancer surgery, mild to moderate frailty does not appear to negatively influence long‐term quality of life, effect on daily activities, or satisfaction with treatment. Frail and non‐frail patients reported similar levels of well‐being and contentment with their care one year post‐operatively.

**What does this paper add to the literature?**

- This paper shows that mild to moderate frailty, assessed using the Clinical Frailty Scale (CFS‐9), does not negatively affect quality of life, daily functioning, or treatment satisfaction one year after colon cancer surgery in older adults.

## INTRODUCTION

Colon cancer is common among older adults, with a median age at diagnosis of 75 years in Sweden [1]. Although the incidence among younger individuals is rising, colon cancer remains strongly age‐associated, and advancing age is the most significant risk factor for its development [2, 3]. Curative treatment typically involves segmental resection surgery [2, 4]. Advanced age is associated with an increased risk of postoperative mortality, complications, and functional decline, including a greater need for assistance with activities of daily living (ADL) and reduced motility after colon cancer surgery [5, 6, 7].

Frailty assessment helps identify individuals with reduced physiological reserve and is an important tool for better reflecting biological, rather than chronological, age [8, 9]. The prevalence of frailty increases with age, and frailty itself is an independent risk factor for postoperative mortality and morbidity in patients undergoing colon cancer surgery [10, 11, 12, 13, 14]. These risks appear to be most pronounced in the immediate postoperative period, with the adverse effects of CFS‐9 assessed frailty diminishing over time [15]. Furthermore, frailty has been associated with poorer overall quality of life [16] as well as more severe symptoms and poorer health‐related quality of life (HRQoL) following cancer treatment across various diagnoses [17].

Given that frailty is strongly associated with impaired postoperative outcomes following colorectal cancer surgery, treatment decision‐making in older adults living with frailty can be challenging. Furthermore, the patients' own perspective of which outcomes matter most to them after surgery remains insufficiently understood [18]. Previous studies have shown that maintaining quality of life (QoL), avoiding functional decline and preserving independence may, for some frail older individuals, be considered more important than survival at any cost, in the context of cancer treatment [19, 20, 21, 22].

There are studies reporting improvements in both short‐ and long‐term QoL among frail older adults after colorectal cancer surgery [21, 23], findings that align with research of older adults with other health conditions [24, 25]. On the other hand, existing evidence suggests that long‐term functional dependence, including reduced activities of daily living (ADL), is common among older patients following colorectal cancer surgery [6, 26, 27].

The aim of this study was to compare self‐assessed QoL and treatment‐related effects on daily activities between frail and non‐frail patients aged ≥70 years one year after colon cancer surgery. In addition, we assessed patient satisfaction with treatment at the one‐year follow‐up. These analyses were based on patient‐reported outcomes from the Quality of Life in Colon Cancer (QoLiCOL) study, an observational, multicentre study investigating postoperative QoL and functional outcomes in patients with colon cancer.

## METHODS

### Study design and data sources

This observational study is based on data from the QoLiCOL study, a prospective, longitudinal, multicentre study conducted between 2015 and 2019 [28]. Patients were recruited from 21 hospitals in Sweden and Denmark. All patients with newly diagnosed colon cancer were eligible and were invited to participate during an outpatient visit at the Department of Colorectal Surgery at any of the recruiting sites. Exclusion criteria were age < 18 years, cognitive impairment that restricted answering questionnaires, and inability to read or write Swedish or Danish. The follow‐up time was three years and questionnaires were completed at diagnosis and at 12 and 36 months. The QoLiCOL study was registered at clinicaltrials.gov (NCT02530593) and was approved by the Regional Ethical Review Board in Gothenburg, Sweden (registration no. EPN 957‐14), and by the regional ethics committee in Denmark (registration no. H‐16027323). Results from other sub‐studies within the QoLiCOL study have been published previously [29, 30].

The present study includes patients aged ≥70 years who underwent colon cancer resection at one of six participating centres (both county and university hospitals) in Region Västra Götaland (VGR), Sweden, and who completed both the baseline and 1‐year QoLiCOL questionnaires. Region Västra Götaland is one of the largest health care regions in Sweden and serves approximately 1.7 million residents. Access to medical records required for the frailty assessment was limited to patients treated within VGR; consequently, patients included outside this region were excluded from the present analysis. This sub‐study was approved by the Swedish Ethical Review Authority (Dnr 2025‐03568‐02).

Baseline data were obtained from the prospective QoLiCOL cohort study, including self‐reported questionnaire data, as well as clinical information from the national quality registry, the Swedish Colorectal Cancer Registry (SCRCR). Directed acyclic graphs (DAGs) were pre‐specified for the relationships between baseline variables and each outcome variable, identifying confounders and mediators (DAGs are available in the supplementary files, Figures S1–S3).

### Questionnaires

The QoLiCOL questionnaires consist of approximately 250 items designed to capture a wide range of symptoms and patient experiences, including QoL and dysfunctions. The questionnaires were developed based on semi‐structured in‐depth interviews and previously validated questions [31]. For the present study, a selected subset of items related to postoperative QoL, effects on daily activities and satisfaction with the received treatment were analysed. Baseline questionnaires were distributed at the time of diagnosis and completed prior to surgery.

### Frailty assessment

A retrospective medical record review was conducted for all eligible patients to assess frailty. Frailty assessment was completed before any follow‐up questionnaire data were accessed, and therefore was blinded to all outcome variables. All assessments were conducted by a single assessor who received thorough training in the use of CFS‐9 by a national expert in CFS assessments [32]. When the assessor was uncertain, a consensus discussion was held within the study group; consensus was reached in all but two cases. The available information in these two cases was insufficient to make a reliable frailty assessment.

Examples of relevant information used for the assessments include previous medical history, current medications, recent healthcare contacts, findings from physical examination, housing situation, use of informal and formal support services, gait aids and ADL function. The assessments were made on relevant information recorded in the medical records during a time period of two weeks from diagnosis until time of surgery, corresponding to a preoperative frailty assessment.

Each subject was assigned a frailty score ranging from 1 to 9, using the 9‐point Clinical Frailty Scale (CFS‐9). Individuals with a score of four or higher were classified as frail, as this aligns with the most recent version of CFS‐9 (v 2.0) where a CFS‐9 score of 4 is defined as ‘living with very mild frailty’ [33]. The CFS‐9 is based on the accumulation of deficits model [33], originally developed for clinical assessment but also validated for retrospective medical chart review in several recent studies [34, 35, 36, 37].

### Comorbidities

The baseline questionnaire included a comorbidity item in which patients indicated whether they had experienced any of 22 specified medical conditions in the past year. For the current analysis, the study group defined a subset of conditions deemed clinically relevant: stroke, neurological diseases, ischaemic heart disease or heart failure, intermittent claudication, pulmonary disease, kidney disease, liver disease, depression, dementia, joint disorders and diabetes. Subjects who entered having one or more of these conditions within the preceding year were classified as having relevant comorbidity.

### Outcomes

#### Primary objective

Self‐reported quality of life (QoL) at one year was assessed by the question: ‘*How would you like to describe your quality of life during the past month’*. Responses ranged from ‘No quality of life’ to ‘Best possible quality of life’ on a seven‐point Likert scale.

#### Secondary objectives

To investigate differences in perceived effects of treatment, or consequences of treatment, on daily activities, the question: ‘*Has the treatment for your colon cancer, or the effects of the treatment (surgery, chemotherapy, or other treatment), affected your daily activities during the past month?’* was used. Response options: ‘No’, ‘Yes, a little’, ‘Yes, moderately’, and ‘Yes, a lot’.

Furthermore, the question ‘*Are you satisfied with the treatment for your colon cancer?*’ was used to assess contentment with the received treatment. The question had three possible answers: ‘Yes’, ‘No’ and ‘Don't know’. Responses of ‘Don't know’ were excluded from the analysis. All outcome variables were compared between the groups non‐frail (CFS < 4) and frail (≥4).

### Statistical analyses

A statistical analysis plan was finalised before analysis. Baseline variables were compared using χ^2^‐ or Fisher's exact test. Ordinal logistic regression (OLR) was used for the outcomes *QoL* and ‘*treatment‐related effect on daily activities*’, due to the variables being ordinal with more than two response categories. Results are reported as odds ratios (OR) with 95% confidence intervals. The proportional odds assumption was evaluated by dichotomising each ordinal outcome at all possible thresholds (e.g., QoL 1 vs. 2–6, QoL 1–2 vs. 3–6, etc.) and comparing effect estimates across binary logistic regression models. Non‐overlapping confidence intervals were interpreted as evidence of violation of proportional odds. The binary endpoint ‘content with treatment’ was analysed using logistic regression, reported as OR.

All analyses were performed using two alternative approaches: direct effect analysis (adjusted for confounders and mediators) and total effect analysis (adjusted for confounders only). The selection of confounders and mediators was based on the pre‐specified DAGs.

### Missing data

Missing data were handled using multiple imputation by chained equations (MICE), 50 imputations with 10 iterations were used. The full set of adjustment factors specified in the SAP, together with the relevant outcomes, was used as an imputation model.

## RESULTS

### Patient characteristics

**FIGURE 1:**
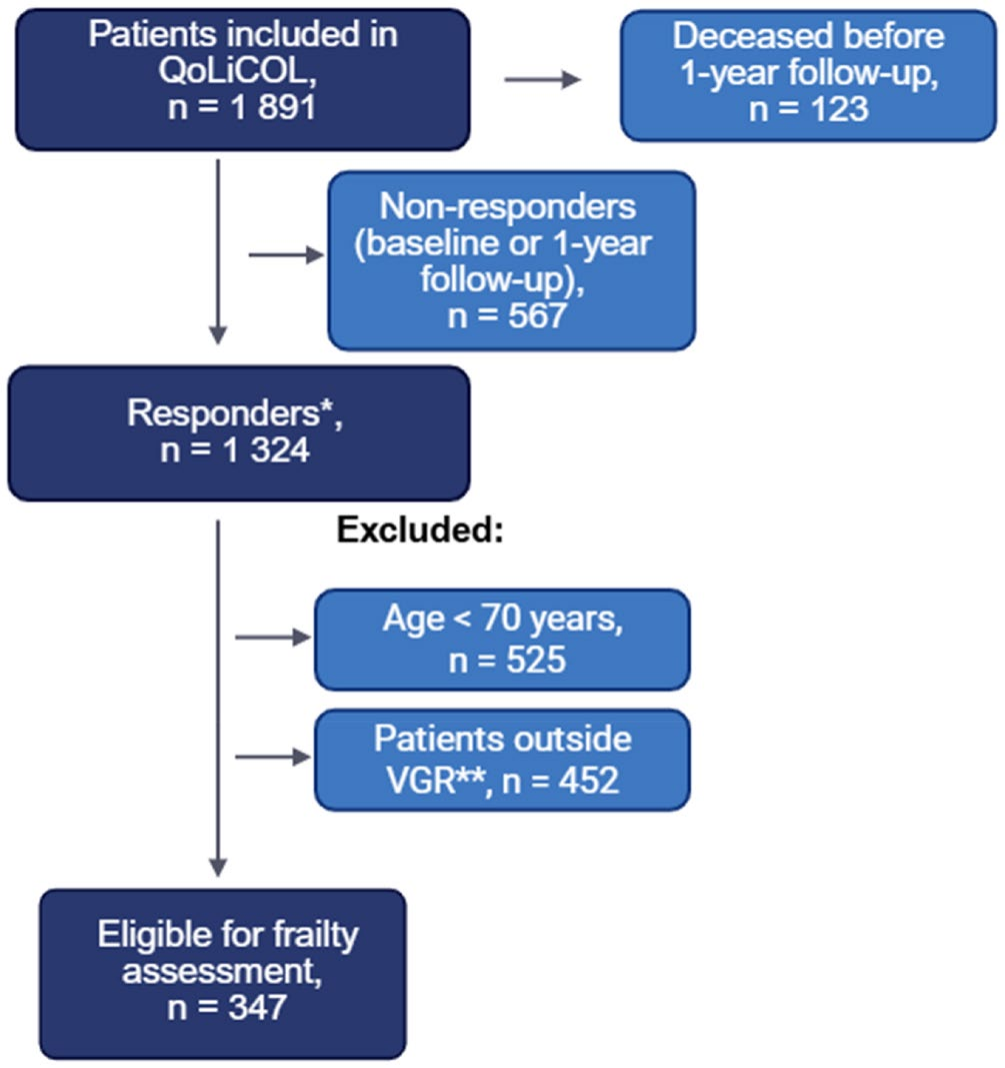
Flow‐chart of study population. Data obtained from the QoLiCOL study. *Responders = participants who completed both baseline‐ and one‐year questionnaires, **Six centres in VGR recruited patients in the QoLiCOL study. VGR, Region Västra Götaland.

A total of 1,891 patients were included in the QoLiCOL study, of whom 123 patients died within the first 12 months of follow‐up. Of the surviving participants, 1324 completed the questionnaires at both baseline and 1‐year follow‐up. Previous studies have presented the clinical characteristics of non‐responders, showing a similar distribution of sex, age and treatment intents, but higher ASA classification and slightly more advanced tumour stage among non‐responders [29]. In the current study, only patients aged ≥70 who underwent surgery at participating hospitals in Region Västra Götaland were included; therefore, younger patients (*n* = 525) and patients who had resection surgery elsewhere (*n* = 452) were excluded, resulting in a cohort of 347 patients (Figure 1). Frailty assessments could not be completed for two individuals.

**FIGURE 2:**
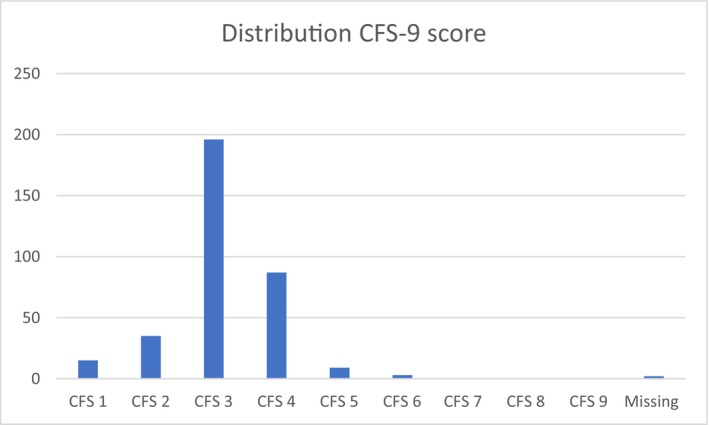
Distribution of Clinical Frailty Scale (CFS‐9 scores in the entire study cohort.

Baseline characteristics of the study population are presented in Table 1. The prevalence of frailty, defined as CFS‐9 score ≥ 4, was 29%. The median CFS‐9 score was three in the non‐frail group (CFS 1–3) and four in the frail group (CFS ≥4); the distribution of CFS‐9 scores in the entire cohort is displayed in Figure 2. Baseline characteristics were similarly distributed between the two groups for age, sex, BMI, and stage according to cTNM. However, a larger proportion of frail patients were classified as ASA III (48.5% vs. 22.0%, *p* < 0.001), and relevant comorbidities were more common in the frail group (64.6% vs. 49.2%, *p =* 0.02). Frail patients reported lower baseline QoL (median 3, range 1–6) compared with non‐frail patients (median 4, range 1–6).

**TABLE 1:** Patient characteristics for the study population, tabulated by CFS‐9 (<4 vs ≥4).

Variables	Non‐frail	Frail	Overall
	(*n* = 246)	(*n* = 99)	(*n* = 347)
Age			
	76.0 [70.0, 95.0]	78.0 [70.0, 91.0]	77.0 [70.0, 95.0]
Sex			
Male	129 (52.4)	50 (50.5)	181 (52.2)
Female	50 (47.6)	49 (49.5)	166 (47.8)
BMI			
	25.2 [15.6, 42.2]	26.6 [16.7, 45.7]	25.3 [15.6, 45.7]
Missing	6 (2.4)	7 (7.1)	15 (4.3)
Stage (cTNM)			
I	61 (24.8)	20 (20.2)	81 (23.3)
II	56 (22.8)	28 (28.3)	84 (24.2)
III	69 (28.0)	26 (26.3)	95 (27.4)
IV	17 (6.9)	6 (6.1)	23 (6.6)
Missing	43 (17.5)	19 (19.2)	64 (18.4)
ASA classification			
1	20 (8.1)	8 (8.1)	28 (8.1)
2	158 (64.2)	37 (37.4)	195 (56.2)
3	54 (22.0)	48 (48.5)	102 (29.4)
4–5	1 (0)	0 (0)	0 (0)
Missing	13 (5.3)	6 (6.1)	21 (6.1)
Relevant comorbidity			
No	125 (50.8)	35 (35.4)	161 (46.4)
Yes	121 (49.2)	64 (64.6)	186 (53.6)
Adjuvant treatment			
No	168 (68.3)	69 (69.7)	237 (68.3)
Yes	73 (29.7)	26 (26.3)	99 (28.5)
Missing	5 (2.0)	4 (4.0)	11 (3.2)
CFS‐9 score at baseline			
	3 [1, 3]	4 [4, 6]	3 [1, 6]
Missing	–	–	2 (0,6)
QoL at baseline			
	4 [1, 6]	3 [1, 6]	4 [1, 6]
Missing	6 (2.4)	2 (2.0)	8 (2.3)

*Note*: Baseline variables presented for all subjects. The values are expressed as median [min, max] or number (%). QoL was assessed using a seven‐point scale ranging from 0 (no quality of life) to 6 (best possible quality of life).

Abbreviations: ASA, American Society of Anaesthesiologists; BMI, body mass index; CFS‐9, Clinical Frailty Scale‐9; TNM, tumour node metastasis; QoL, quality of life.

### Outcome variables

For the outcomes *QoL* and *treatment‐related effect on daily activities*, at the 1‐year follow‐up, ordinal logistic regression (OLR) was used under the proportional odds assumption. As described in the methods section, a series of binary logistic regression models was fitted for the endpoints. The confidence intervals for the estimated effects overlapped across all cut‐points, indicating no evidence of a violation of the proportional odds assumption.

No significant differences were observed between frail and non‐frail patients in QoL, treatment‐related effect on daily activities during the last month, or contentment with treatment at the 1‐year follow‐up (Table 2). Only a small proportion of participants reported not being content with treatment (*n* = 8; 2%).

**TABLE 2:** Outcome variables at 12‐month follow‐up, tabulated by CFS‐9 (<4 vs ≥4).

Variable	Non‐frail	Frail	Overall	OR (95% CI)	*p*‐value
QoL the last month	5 [0, 6]	5 [2, 6]	5 [0, 6]	0.86 (0.54–1.35)	0.50
Missing	6 (2.4)	2 (2.0)	8 (2.3)		
Treatment‐related effect on daily activities				1.23 (0.7–2.12)	0.48
No	186 (75.6)	64 (64.6)	252 (72.6)		
Yes, a little	37 (15.0)	16 (16.2)	53 (15.3)		
Yes, moderately	15 (6.1)	11 (11.1)	26 (7.5)		
Yes, a lot	5 (2.0)	4 (4.0)	9 (2.6)		
Missing	3 (1.2)	4 (4.0)	7 (2.0)		
Content with treatment				2.35 (0.41–14.0)	0.32
No	6 (2.4)	2 (2.0)	8 (2.3)		
Yes	230 (93.5)	90 (90.9)	322 (92.8)		
Don't know	8 (3.3)	6 (6.1)	14 (4.0)		
Missing	2 (0.8)	1 (1.0)	3 (0.9)		

*Note*: Outcome variables are reported for all subjects. QoL was assessed using a six‐point scale ranging from 0 (no quality of life) to 6 (best possible quality of life). Values are presented as median [min, max] or number (%). Results for QoL and ‘treatment‐related effect on daily activities’ are derived from the total effect OLR analysis comparing non‐frail and frail groups, adjusted for confounders (age, comorbidity and ASA) but not for potential mediators. Results for ‘content with treatment’ are derived from total effect logistic regression comparing non‐frail and frail groups, adjusted for confounders (comorbidity and ASA) but not for potential mediators.

Abbreviations: ASA, American Society of Anaesthesiologists; CI, confidence interval; OLR, ordinal logistic regression; OR, odds ratio; QoL, quality of life.

Both frail and non‐frail patients reported improved self‐assessed QoL at the 1‐year follow‐up (median 5 (range 0–6) vs. 5 (range 2–6)) compared with baseline (median 3 (range 1–6) vs. 4 (range 1–6)). The results were similar, without significant differences between groups, even when the cut‐off for frailty was set at a CFS‐9 score of 5 (data not shown). Full model results for both the total effect primary analyses and the direct effect analyses of primary and secondary outcomes can be found in the supplementary files (Tables S1–S5).

## DISCUSSION

### Main findings

This study indicates that frail older adults tend to maintain or even improve their QoL in the long term after curative colon cancer surgery. Notably, frail patients did not report a greater long‐term impact of cancer treatment on their ability to perform daily activities or on self‐assessed QoL compared with non‐frail patients. Taken together, these results suggest that concerns about recommending surgery for mildly to moderately frail patients, based on assumptions that treatment may lead to long‐term reductions in QoL or increased functional dependence, may be overstated or not universally applicable.

### Strengths

A major strength of this study is its explicit focus on frailty rather than chronological age as a risk marker. Although colon cancer is a common and extensively studied condition, relatively few studies have examined long‐term functional outcomes after colon cancer surgery [6]. Moreover, most previous studies have been limited by small sample sizes [23] and have focused primarily on chronological age rather than frailty [21], potentially limiting their ability to capture the true impact of frailty on postoperative recovery. By incorporating frailty assessment and patient‐reported outcomes, this study provides clinically relevant information with the potential to improve shared decision‐making for frail older adults.

Additional strengths include the long‐term follow‐up and the relatively large cohort size compared with previous studies in this field. The inclusion of self‐reported QoL and treatment‐related impact on daily living captures outcomes that are highly relevant to older patients and may not be adequately reflected by traditional clinical endpoints alone. Furthermore, data were collected from six participating centres within a large Swedish region, thereby increasing the robustness and generalisability of the findings.

### Limitations

Several limitations should be acknowledged. First, the prevalence of frailty in the study cohort was 29%. In previous work from our research group, using the same assessment method in patients aged ≥70 years undergoing colorectal cancer surgery in the same healthcare region and during an overlapping period, the prevalence of frailty was 56% [13]. International studies have estimated the frailty prevalence among older adults (aged ≥65 years) with colorectal cancer to be in the range of 25%–46% [10]. The lower‐than‐expected prevalence in this material is likely multifactorial but may partly reflect selection bias. Cognitive impairment, that restricted answering questionnaires, was an exclusion criterion in the QoLiCOL study, and individuals with cognitive impairment often exhibit more advanced frailty when assessed using the Clinical Frailty Scale. In addition, frail individuals may have been more reluctant or less able to complete questionnaires, resulting in their underrepresentation.

These assumptions are supported by the fact that only very mildly to moderately frail patients (CFS‐9 scores 4–6) were included, indicating that individuals with more advanced frailty were not captured. Moreover, the risk of postoperative mortality increases with each incremental step on the CFS‐9 [13], and patients with more advanced frailty may therefore have died to a greater extent during the one‐year follow‐up, resulting in their absence from the final dataset. Consequently, the findings cannot be readily extrapolated to patients with more advanced frailty. However, clinical decision‐making in this latter group is often more straightforward, given their substantially reduced functional reserve and clearly elevated perioperative risks. This study provides new insights into patient‐reported outcomes among the large group of mildly to moderately frail older adults, a group where clinical decision‐making can be more challenging.

Another potential limitation is the retrospective frailty assessment, which relied solely on information available in medical records. Although measures were taken to enhance the validity of the assessments, the retrospective approach may have limited the accurate identification of all levels of frailty. The Clinical Frailty Scale is most informative when used as a granular measure across the whole spectrum [33, 38], and the restricted range in this cohort prevented analyses of heterogeneity within the frail group. Furthermore, comorbidity data were based on patient‐reported information rather than medical records, which could be associated with some degree of uncertainty in the assessment of comorbidities.

Regarding the analysed item ‘*are you content with the treatment of your colon cancer*’ it should be noted that only a very small proportion of participants reported not being content (eight individuals, 2%). Consequently, these analyses are likely underpowered to detect any effect of frailty on contentment with treatment. Nevertheless, it is notable that 98% of older adults in this cohort were content with their received treatment long term.

Finally, the dataset did not contain information regarding postoperative complications, stoma status, or other potentially limiting factors that may have influenced patient‐reported QoL or functional dependence. It can be assumed that frail patients were more likely to receive a stoma or experience postoperative complications compared with non‐frail patients. Despite this, the self‐reported outcome measures in this study were comparable between the two groups.

### Clinical implications and future perspectives

The results from the present study align with previous observations indicating that the negative impact of frailty at the time of surgery on outcomes after colon cancer surgery tends to diminish over time. In the context of shared decision‐making, where patients and their relatives are actively involved, the healthcare team must work to identify and understand what each patient values and fears most. Maintaining independence and preserving QoL are important to all patients but may hold particular importance for frail individuals who already experience greater dependency and symptom burden in daily life [19, 20, 21]. This suggests that future efforts should focus on reducing early postoperative risks. If measures are taken to improve survival and avoid serious complications in the immediate postoperative period for frail older adults, existing evidence [15], including the results from this study, indicates that their long‐term prognosis, including QoL and functional capacity, may be comparable to that of non‐frail patients.

## AUTHOR CONTRIBUTIONS

**Maria Normann:** Conceptualisation; Methodology; Investigation; Data curation; Writing—original draft. **Eva Haglind:** Methodology; Writing—review and editing. **Peter Matthiessen:** Methodology; Writing–review and editing. **Jacob Rosenberg:** Methodology; Writing—review and editing. **Niklas Ekerstad:** Conceptualisation; Methodology; Investigation; Writing—review and editing. **Eva Angenete:** Conceptualisation; Methodology; Investigation; Writing—review and editing. **Mattias Prytz:** Conceptualisation; Methodology; Investigation; Data curation; Writing—review and editing. All authors read and approved the final manuscript.

## FUNDING INFORMATION

The study is supported by the Department of Research and Development Västra Götalandsregionen (VGFOUREG‐981985, VGRFOU‐773172, VGRFOU‐557431, VGRFOU‐64444). Mary von Sydows stiftelse. Cancerfonden, CAN 2016/509, 190333 nr 222265. Lions Cancerfond Väst, 2017:30. Svenska Läkarsällskapet, SLS‐693371. Vetenskapsrådet 201‐01103, 2021‐01025. ALF (Swedish organisation). ALFGBG‐493341, AFLGBG‐716581, AFLGBG‐965084. The funding parties have no role in the design of the study or collection, analysis, or interpretation of data, nor in the writing of the manuscript.

## CONFLICT OF INTEREST STATEMENT

The authors declare they have no competing interests.

## ETHICS STATEMENT

The QoLiCOL study was approved by the Regional Ethical Review Board in Gothenburg, Sweden (registration no. EPN 957–14), and by the regional ethics committee in Denmark (registration no. H‐16027323). This sub‐study was approved by the Swedish Ethical Review Authority (Dnr 2025‐03568‐02).

## TRIAL REGISTRATION

The QoLiCOL study was registered at clinicaltrials.gov (NCT02530593).

## Supporting information


**Table S1.** The results of the total effect primary analysis, impact of frailty on QoL at 12 months.
**Table S2**. The results of the direct effect analysis of primary outcome QoL at 12 months.
**Table S3**. The results of the total effect analysis regarding the secondary outcome, impact of Frailty on treatment‐related effect on ADL at 12 months.
**Table S4**. The results of the direct effect analysis of the secondary outcome, treatment‐related effect on ADL (12 month).
**Table S5**. The results of the total effect analysis of contentment with treatment.

**Figure S1.** DAG displaying the relationship between the exposure ‘Frailty’ and the primary endpoint ‘Quality of Life’ at one year postoperatively. Relevant covariates described in figure.
**Figure S2**. DAG displaying the relationship between the exposure ‘Frailty’ and the secondary objective ‘Treatment effect on ADL’ at one year postoperatively.
**Figure S3**. DAG displaying the relationship between the exposure ‘Frailty’ and the secondary outcome variable ‘Content with treatment’ at one year postoperatively.

## Data Availability

Anonymised data are available from the corresponding author upon reasonable request.
