# Supplementary material for: Mild to moderate frailty among older adults does not affect long‐term quality of life or functional outcomes after colon cancer surgery
Source: Colorectal Dis. 2026 Apr 7;28(4):e70438. doi: 10.1111/codi.70438 (PMC13054916; doi:10.1111/codi.70438)

Table S1. The results of the total effect primary analysis, impact of frailty on QoL at 12 months.


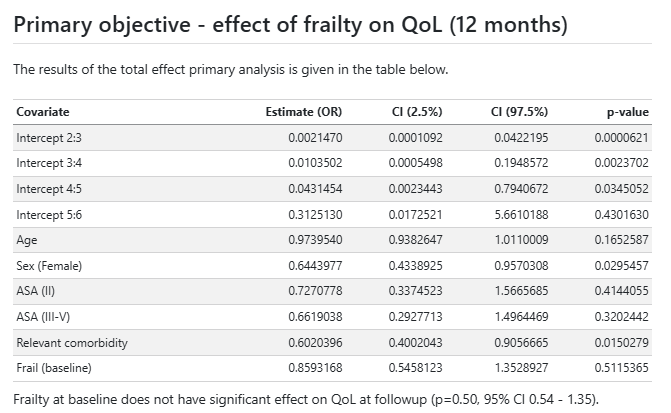


Table S2. The results of the direct effect analysis of primary outcome QoL at 12 months.


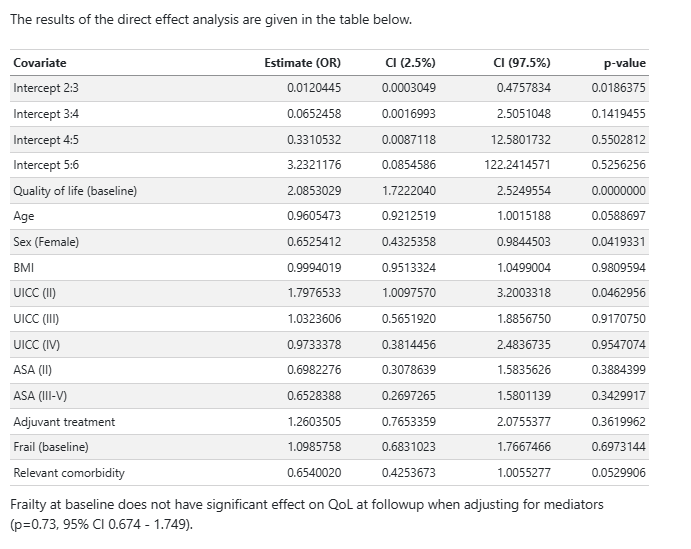


Table S3. The results of the total effect analysis regarding the secondary outcome, impact of Frailty on treatment-related effect on ADL at 12 months


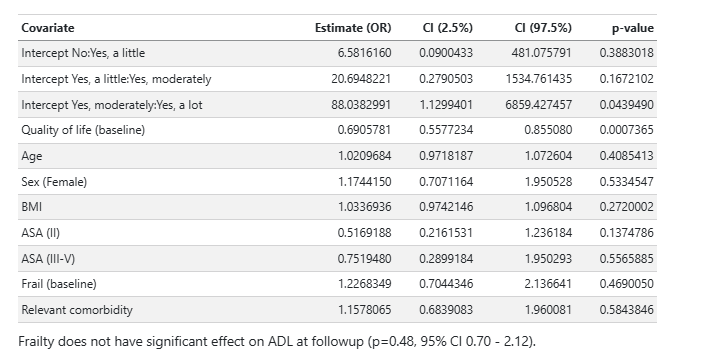


Table S4. The results of the direct effect analysis of the secondary outcome, treatment-related effect on ADL (12 month).


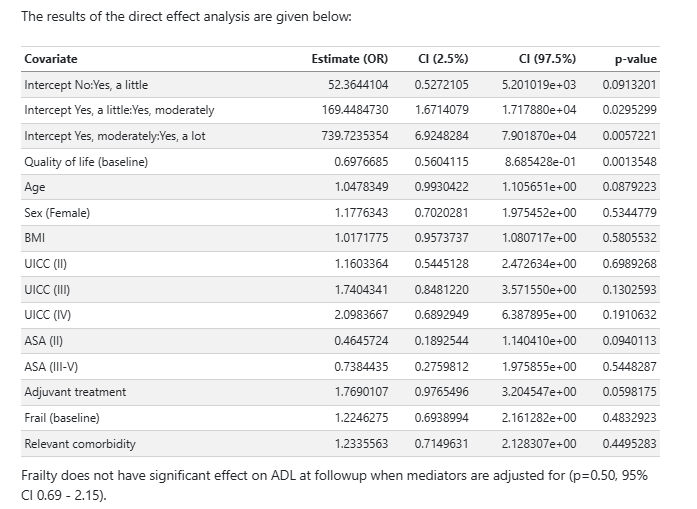


Table S5. The results of the total effect analysis of contentment with treatment.


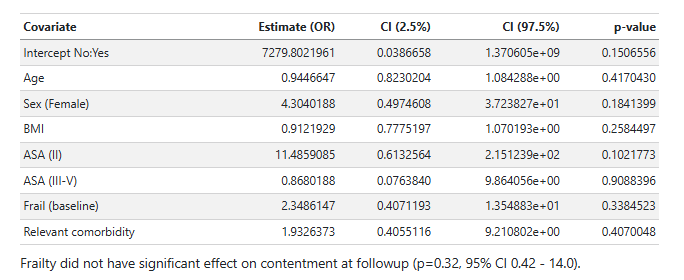


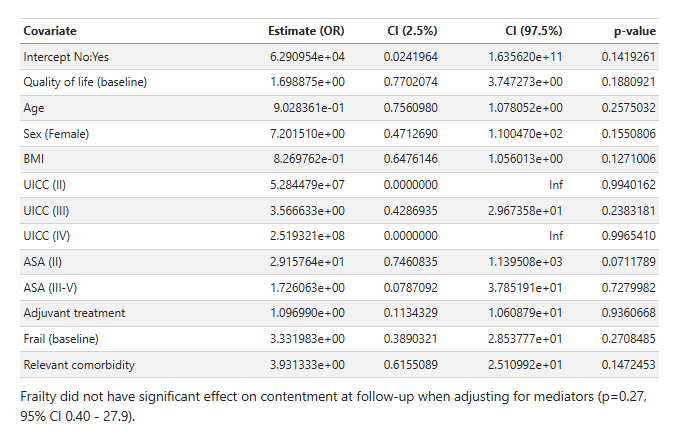

Supplement: Supplementary file 1 — Table S1. The results of the total effect primary analysis, impact of frailty on QoL at 12 months. Table S2. The results of the direct effect analysis of primary outcome QoL at 12 months. Table S3. The results of the total effect analysis regarding the secondary outcome, impact of Frailty on treatment‐related effect on ADL at 12 months. Table S4. The results of the direct effect analysis of the secondary outcome, treatment‐related effect on ADL (12 month). Table S5. The results of the total effect analysis of contentment with treatment. [file CODI-28-0-s001.docx]
