# Supplementary material for: Mild to moderate frailty among older adults does not affect long‐term quality of life or functional outcomes after colon cancer surgery
Source: Colorectal Dis. 2026 Apr 7;28(4):e70438. doi: 10.1111/codi.70438 (PMC13054916; doi:10.1111/codi.70438)
Supplement: Supplementary file 2 — Figure S1. DAG displaying the relationship between the exposure ‘Frailty’ and the primary endpoint ‘Quality of Life’ at one year postoperatively. Relevant covariates described in figure. Figure S2. DAG displaying the relationship between the exposure ‘Frailty’ and the secondary objective ‘Treatment effect on ADL’ at one year postoperatively. Figure S3. DAG displaying the relationship between the exposure ‘Frailty’ and the secondary outcome variable ‘Content with treatment’ at one year postoperatively. [file CODI-28-0-s002.docx]

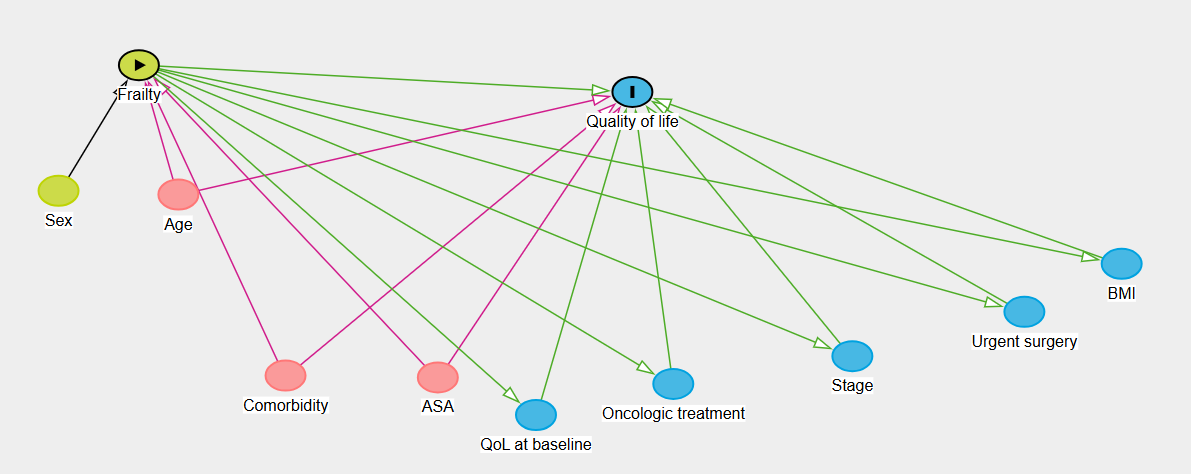


Figure S1. DAG displaying the relationship between the exposure “Frailty” and the primary endpoint “Quality of Life” at one year postoperatively. Relevant covariates described in figure.


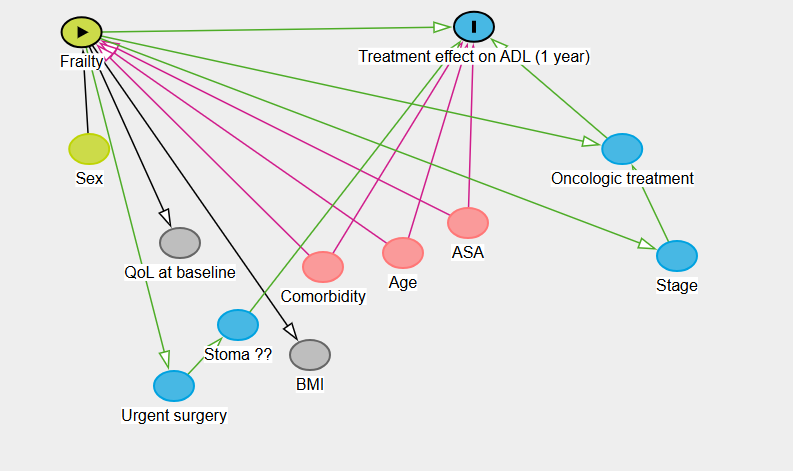


Figure S2. DAG displaying the relationship between the exposure “Frailty” and the secondary objective “Treatment effect on ADL” at one year postoperatively.


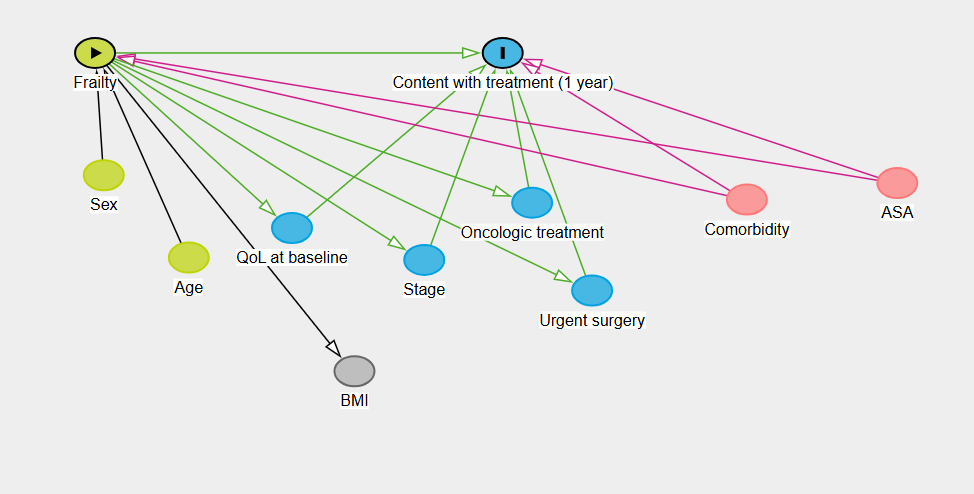


Figure S3. DAG displaying the relationship between the exposure “Frailty” and the secondary outcome variable “Content with treatment” at one year postoperatively.
